# Formation of Sclerotia and Production of Indoloterpenes by *Aspergillus niger* and Other Species in Section *Nigri*

**DOI:** 10.1371/journal.pone.0094857

**Published:** 2014-04-15

**Authors:** Jens C. Frisvad, Lene M. Petersen, E. Kirstine Lyhne, Thomas O. Larsen

**Affiliations:** Chemodiversity Group, Department of Systems Biology, Technical University of Denmark, Lyngby, Denmark; University of Missouri, United States of America

## Abstract

Several species in *Aspergillus* section *Nigri* have been reported to produce sclerotia on well-known growth media, such as Czapek yeast autolysate (CYA) agar, with sclerotia considered to be an important prerequisite for sexual development. However *Aspergillus niger sensu stricto* has not been reported to produce sclerotia, and is thought to be a purely asexual organism. Here we report, for the first time, the production of sclerotia by certain strains of *Aspergillus niger* when grown on CYA agar with raisins, or on other fruits or on rice. Up to 11 apolar indoloterpenes of the aflavinine type were detected by liquid chromatography and diode array and mass spectrometric detection where sclerotia were formed, including 10,23-dihydro-24,25-dehydroaflavinine. Sclerotium induction can thus be a way of inducing the production of new secondary metabolites from previously silent gene clusters. Cultivation of other species of the black aspergilli showed that raisins induced sclerotium formation by *A. brasiliensis*, *A. floridensis A. ibericus*, *A. luchuensis*, *A. neoniger*, *A. trinidadensis* and *A. saccharolyticus* for the first time.

## Introduction

Certain strains in several species of Aspergillus section Nigri have been reported to produce sclerotia, notably Aspergillus sclerotioniger, A. carbonarius, A. tubingensis, A. sclerotiicarbonarius, A. costaricaensis, A. ellipticus, A. japonicus, A. piperis, A. aculeatus, A. aculeatinus, A. brunneoviolaceus and A. violaceofuscus [1]–[9]. Some species were originally described as producers of sclerotia, for example A. heteromorphus [10]. However Al-Musallam [3] could not induce sclerotium production in this species, despite several attempts. A. indologenus (as A. aculeatus CBS 114.80) was reported as a producer of abundant sclerotia [3], but Samson et al. [6] did not observe sclerotia in that strain. Concerning Aspergillus niger, only a few strains have been reported to produce sclerotia, WB 346 [1], [11]–[12], WB 5121 = CBS 553.65 [1], CBS 425.65 [3] and WB 4700 [13]. However these strains represent other species than Aspergillus niger sensu stricto, for example CBS 553.65 was allocated to Aspergillus costaricanesis by Samson et al. [6] and WB 346 is representative of Sterigmatocystis fusca Bain., now regarded as Aspergillus carbonarius [3], CBS 425.65 is an A. tubingensis [14] and WB 4700 is also an A. tubingensis [13]. Sclerotium-producing strains from Tübingen and other German cities, identified as A. niger [15], are probably also A. tubingensis.

Genome sequencing projects [16], [17], [18] have shown the presence of predicted secondary metabolite gene clusters with no known expression and some of these could code for strictly sclerotium-associated metabolites such as aflavininins, aflavarins and aflatrems in *Aspergillus flavus* sclerotia [19] Sclerotium-producing strains in section *Nigri* that have been examined produced many sclerotium-borne secondary metabolites. *Aspergillus tubingensis* NRRL 4700 was reported to produce tubingensin A & B, dihydrotubingensis A & B, 14-epi-14-hydroxy-10,23-dihydro-24,25-dehydroaflavinine, 10,23-dihydro-24,25-dehydroaflavinine and 10,23-dihydro-24,25-dehydro-21-oxo-aflavinine [20]–[23]. In order to induce sclerotium production in *Aspergillus carbonarius*, Raper and Fennell [1] stated that steep agar, Czapek agar containing corn steep liquor, was a most suitable medium. Indeed *A. carbonarius* produced large amounts of sclerotia containing ochratoxin A on corn kernels [24]. Al-Musallam [3] reported that *A. tubingensis* CBS 425.65 produced sclerotia only when grown on French beans, while *A. tubingensis* WB 4700 produces sclerotia freely on most media [1], [13]. This shows that sclerotium production may be strain-specific, but can be induced by plants parts such as beans, corn kernels in those isolates that do not produce them on standard identification media such as those based on Czapek, malt extract or oat meal.

Sclerotial development is considered to be an important prerequisite for sexual development in *Aspergillus* section *Circumdati*, and sclerotia might also have a role in dormancy [25]. Since a sexual state has been found in species in closely related sections of *Aspergillus*, i.e. in *A. flavus*, *A. parasiticus* and *A. nomius* in *Aspergillus* section *Flavi* [26]–[28] and *A. muricatus* [29] in *Aspergillus* section *Circumdati* [30] the ability to induce sclerotium production could be a first step towards finding a perfect state in *Aspergillus niger*. Rajendran and Muthappa [2] showed that *Aspergillus japonicus* has a perfect state and is a homothallic species with cleistothecia formed in one or two cavities (loculi) in sclerotia. This species was named *Saitoa japonica*. Later *A. sclerotiicarbonarius* [31] and *A. tubingensis* [32] were found to be heterothallic. These species also produced cleistothecia within one or two loculi within the sclerotia [31]–[32]. Some strains from section *Nigri* produce sclerotia on commonly used media, so Horn et al. [32] were able to induce the perfect state in *Aspergillus tubingensis* after determining the mating types of sclerotial strains and cross them on mixed cereal medium. *A. niger* is closely related to *A. tubingensis* and is probably also heterothallic, but the lack of sclerotia in *A. niger* hinders the test of whether a perfect state can be induced in this important industrial species [33]. *A. niger* is a very common spoilage organism on grapes, dried fruits, peanuts, maize, coffee and many other plants, so we wanted to test whether any of these fruits could induce sclerotium production in *A. niger* and other species in *Aspergillus* section *Nigri*. We also hypothesized that by inducing sclerotium production we could activate silent gene clusters for fungal indolo-terpenes, and thereby find new secondary metabolites in these fungi.

Even though many secondary metabolites have been reported from *Aspergillus niger*, this species has never been reported to produce aflavinin related indoloterpenes [34]. It is known, however, that sclerotium production may be associated with increased ochratoxin A production, at least in *A. carbonarius* [24], and other sclerotium producing species such as *A. sclerotioniger* also produce ochratoxin A [5]-[6], [8]. The species in the *A. carbonarius* clade have been reported to only produce ochratoxin A, and no aflavinines, whereas *A. tubingensis* and *A. costaricaensis* produce aflavinins in the sclerotia [6], [22] and no ochratoxins. If *A. niger* produces sclerotia when induced by different fruits and other plant parts they may also produce increased amounts of ochratoxin A, which would have important consequences for food safety. Possible sclerotium production by *A. niger* and other black Aspergilli would have many biological, biotechnological and food safety consequences [33], [35]–[42]. One paper has indicated that *A. niger* might be able to produce sclerotia, as it was found that certain poorly sporulating UV mutants of *A. niger* produced sclerotium-like structures [43]. We wanted to test our hypothesis that natural substrates for fungi might induce sclerotium production and thereby confirm that previously identified “silent” gene clusters do indeed have a functional role in *A. niger* and other species in section *Nigri*.

## Materials and Methods

### Media and fungi

Representatives of all species in *Aspergillus* section *Nigri* were selected with an emphasis on isolates from plants substrates, and identified to species level using a polyphasic approach [5]–[6], [8], [14], [44]–[45]. A series of strains of *Aspergillus niger sensu stricto* and *A. tubingensis* from raisins were isolated from the medium DG18 (dichloran glycerol 18% agar) [46] on which raisins from different countries have been plated out [36]. All black aspergilli were isolated, and pure cultures were identified and accessioned in the IBT collection (Collection of fungal strains at the Department of Systems Biology, Technical University of Denmark, Lyngby, Denmark). *Aspergillus* section *Nigri* strains from other collections were also tested (Table 1 and 2). All strains were 3-point inoculated on the media Czapek yeast autolysate agar (CYA), CYA with three autoclaved black raisins (CYAR) (or other plant parts). During identification the strains were also inoculated on malt extract agar according to Blakeslee (MEA), yeast extract sucrose (YES) agar, creatine-sucrose (CREA) agar, oat-meal agar (OA), and some isolates were also inoculated on Wickerhams antibiotic test medium (WATM), and potato dextrose agar (PDA). All media were poured in 11 cm plastic Petri dishes, inoculated and incubated at 25°C for 7 days in darkness [47]–[48].

**Table 1:** Production, or not, of sclerotia by *Aspergillus niger* strain IBT 29019, IBT 24631, and IBT 24634, and production of apolar indoloterpenes on Czapek yeast autolysate agar with or without added plant parts.

CYA + additional fruit/cereal grain	*Aspergillus niger* strain	Sclerotia produced	10,23-dihydro-24,25-dehydroaflavinine produced	Number of apolar indoloterpenes detected by liquid chromatography diode array detection (LC-DAD)
None	IBT 29019	-*	-	0/0
None	IBT 24631	-	-	0/0
None	IBT 24634	-	-	0/0
Raisin (CYAR)	IBT 29019	++*	+	2/2/2/**4** **
Raisin (CYAR)	IBT 24631	+*	+	1/1/1/**2** **
Raisin (CYAR)	IBT 24634	-	-	0/0
Mulberry	IBT 29019	++	+	2
Mulberry	IBT 24631	-	-	0
Mulberry	IBT 24634	-	-	0
Blueberry	IBT 29019	++	+	2/3
Blueberry	IBT 24631	-	-	0
Blueberry	IBT 24634	-	-	0
Cranberry	IBT 29019	++	+	2/7
Cranberry	IBT 24631	-	-	0
Cranberry	IBT 24634	-	-	0
Goji berry	IBT 29019, 24631, 24634	-	-	0
Apricot	IBT 29109	++	+	2/7
Apricot	IBT 24631	−/+***	+	0/1
Apricot	IBT 24634	-	-	0/0
Prune	IBT 29019	++	+	2/3
Prune	IBT 24631	−/+***	+	0/1
Prune	IBT 24634	-	-	0/0
Mango, 40%	IBT 29019	++	+	3/5
Mango, 40%	IBT 24631, 24634	-	-	0/0
Mango peel	IBT 29019	++	+	2
Papaya, 40% & 4%	IBT 29019	-	-	0/0
Corn	IBT 29019	-	-	0
Corn	IBT 24631	+	+	1
Corn	IBT 24634	-	-	0
White rice, 50%	IBT 29019	++	+	11/12
White rice, 50%	IBT 24631	+	+	2
White rice, 50% or 40%	IBT 24634	-	-	0
Brown rice, 50%	IBT 29019	++	+	13/13
Brown rice, 40%	IBT 29019	+	+	2/1
Brown rice, 50%	IBT 24631	+	+	1
Brown rice, 50%	IBT 24634	-	-	0

*-: no sclerotia produced, +: few scattered sclerotia, ++: many sclerotia (>10) surrounding each piece of fruit, or directly on the rice or corn.

**More indoloterpenes were detected using mass spectrometric detection than diode array detection in this case where both methods were compared. In IBT 29019 on CYAR, 4 recognizable indoloterpenes were detected using LC-mass spectrometric detection (in bold), but only two according to DAD.

***−/+: sclerotia not produced in one experiment, but produced in the other

The conidia were frozen at −18°C for three weeks before inoculation.

**Table 2:** *Aspergillus* section *Nigri*: Production of sclerotia on CYA agar or CYA with raisins (most sclerotia were formed around the raisins on the agar surface, and few sclerotia if any on the raisins themselves, see footnotes).

Species	Isolates	Sclerotia on CYAR	Sclerotia on CYA	Predominantly sclerotial apolar metabolites and/or ochratoxin A
*A. aculeatinus*	CBS 121875 = IBT 29118, IBT 30576	++*	-	Indoloterpenes produced
*A. aculeatus*	IBT 21030, IBT 13519, IBT 32735 = IMI 240698**	++/+++*	-, ++ in sectors for IBT 21030,+++ for IMI 240698	10,23-dihydro-24,25-dehydroaflavinine, an okaramine, paspa***
*A. brasiliensis*	IBT 28177	++	-	10,23-dihydro-24,25-dehydroaflavinine and 4 other aflavinins No apolar sclerotial metabolites detected in other strains
*A. brunneoviolaceus* (*A. fijiensis*)	IBT 13989 = CBS 313.89	++	++	Paspa
*A. carbonarius*	IBT 21089 = NRRL 369, WB 346, IBT 31277, IBT 29172 = IBT 4916 = CBS 117.49 (on mango)	+*	-	Ochratoxin A
*A. costaricaensis*	IBT 23401 = CBS 115574 = ITEM 7555	+++	+	10,23-dihydro-24,25-dehydroaflavinine, aspernomine,12 other aflavinines
*A. ellipticus* ( = *A. helicothrix*)	IBT 29172 = CBS 707.79, IBT 13963 = CBS 677.79	+	-	No apolar sclerotial metabolites detected
*A. eucalypticola*	IBT 29274 = CBS 122172	-	-	No apolar sclerotial metabolites detected
*A. floridensis*	IBT 32546 = NRRL 62478	++	-	10,23-dihydro-24,25-dehydroaflavinine
*A. heteromorphus*	IBT 13691 = CBS 117.55, IBT 14352 = CBS 312.89	+****	-	No apolar sclerotial metabolites detected
*A. homomorphus*	IBT 21893 = CBS 101889	-	-	No apolar sclerotial metabolites detected, several apolar unique extrolites are present
*A. ibericus*	IBT 26612 = CBS 121593	+	-	An aflavinine
*A. indologenus*	CBS 114.80 = IBT 3679	−/(+)*****	-	Mid-polar indoloterpenes indicate that sclerotia could be produced
*A. japonicus*	IBT 5718 = CBS 114.51	-	-	No apolar sclerotial metabolites detected
*A. lacticoffeatus*	IBT 22031 = CBS 101803	-	-	No apolar sclerotial metabolites
*A. luchuensis* (*A. acidus*)	IBT 24821, IBT 24825, CBS 553.65 = IBT 28612	+	-	10,23-dihydro-24,25-dehydroaflavinine, 3 other aflavinins
*A. neoniger*	IBT 30603, IBT 20973	++	-	10,23-dihydro-24,25-dehydroaflavinine, aspernomine, 10 other aflavinin
*A. niger* (the majority of strains did not produce sclerotia, see Table S2 in File S1)	IBT 24631 = CBS 133816, IBT 26389 = NRRL 599, IBT 28998, IBT 28999, IBT 29000, IBT 29001, IBT 29003, IBT 29005, IBT 29006, IBT 29007, IBT 29019 = CBS 133818, IBT 29020	++/+	-	10,23-dihydro-24,25-dehydroaflavinine, 5 other aflavinins
*A. piperis*	IBT 24630 = CBS 112811	+++	+++	10,23-dihydro-24,25-dehydroaflavinine, 11 other aflavinins
*A. sclerotiicarbonarius*	IBT 28362 = CBS 121057	++/+++	++/+++	Paspa (3 different)
*A. saccharolyticus*	IBT 30881, IBT 28231	++	-	Paspa (one)
*A. sclerotioniger*	IBT 22905	+++	+++	Ochratoxin A
*A. trinidadensis*	IBT 32570 = NRRL 62480	++	-	Paspa (one)
*A. tubingensis* (the majority of strains did not produce sclerotia, see Table S2 in File S1)	IBT 23488 = IBT 16833 = CBS 161.79 = NRRL 4700, IBT 20950, IBT 29022, IBT 29557 = CBS 122.35X, CBS 425.65, IBT 29022, IBT 31740	++/for NRRL 4700 +++	−, +++ for NRRL 4700	14-epi-14-hydroxy-10,23-dihydro-24,25-dehydroaflavinine, 10,23-dihydro-24,25-dehydroaflavinine, 10,23-dihydro-24,25-dehydro-21-oxo-aflavinine, tubingensin A & B, dehydrotubingensin A & B, aspernomine, 20 other aflavinines
*A. uvarum*	IBT 26606 = CBS 121591	-	-	No apolar sclerotial metabolites detected
*A. vadensis*	CBS 113365 = CBS 102787 = IBT 24658	-	-	No apolar sclerotial metabolites detected
*A. violaceofuscus*	CBS 115571 = IBT 14708	++	+	Mid-polar sclerotial metabolites detected
*A. welwitchiae* (“*A. awamori*”), (further strains were tested, none produced sclerotia, see Table S2 in File S1)	CBS 139.54	-	-	No apolar sclerotial metabolites detected

*-: no sclerotia formed, +: Few scattered sclerotia (<10), ++: Several (>10) sclerotia surrounding the raisins, +++: numerous sclerotia formed all over the medium, both with and without raisins.

**Homothallic sexual state reported [2], this strain readily produced abundant sclerotia, both with and without raisins.

***Paspa is probably an indoloterpene with a paspalinine chromophore.

****In this species, sclerotia were only produced on the raisins, not on the medium.

*****Abundant sclerotia reported by Musallam [3].

Isolates not producing sclerotia in *A. brasiliensis*, *A. niger*, and *A. welwitschiae* and are listed in Table S2 in File S1. The conidia were frozen at −18°C for three weeks before inoculation.

In the first experiments *A. niger* IBT 29019 and IBT 24631 produced sclerotia on CYA agar with scattered whole raisins. The black conidia used for inoculation were taken directly from silica gel tubes. However when conidia from colonies grown on CYA for a week at 25°C were used for inoculation, the isolates lost the ability to produce sclerotia on CYA with raisins. Since some raisins are treated with sunflower oil, CYA with 1% sunflower oil was also prepared. To possibly enhance sclerotium production in *A. niger* CYA agar was added 1 mL of biotin solution giving a final concentration of 6.4 mg biotin/L medium and raisins added as biotin induced ascoma formation in *Penicillium rubens* [49]–[50]. The *A. niger* strains IBT 29019, 24631 and 24634 were also inoculated on 40% and 50% white and brown rice (in water) in 200 ml Erlenmeyer flasks, on 50% corn kernels, and on 4% and 40% macerated mango or papaya in water with 2% agar added. Since the sunflower oil or biotin treatments did not induce sclerotium formation, experiments were done to compare non-frozen and frozen inoculum in the three strains of *Aspergillus niger*, as freezing of the inoculum had earlier been shown to enhance sclerotium formation in *A. aculeatus* IBT 21030. This freezing-step consistently helped inducing sclerotium production in *A. niger* IBT 29019 and IBT 24631. The black aspergilli tested were therefore pretreated by placing CYA agar slants in Eppendorf tubes with sporulating fungal strains (6 mm agar plugs, one week old) in a −18°C freezer for at least 3 weeks before inoculation on the Petri plates with and without raisins or other plant parts. In the first series of experiments *A. niger* IBT 29019, IBT 24631 and IBT 24634 were tested on a series of different substrates: CYA agar with and without raisins (of different kinds), dried mulberry, dried blueberry, dried cranberry, dried goji berry, dried apricot, dried prune, mango peel, papaya peel, dried green coffee beans, and dried kidney beans, all organically grown (Table S1 in File S1). All substrates were autoclaved for 20 min at 121°C. In the second series of experiments many strains of *A. niger* and *A. welwitchiae* were tested for sclerotium production in addition to representatives of all other known species in *Aspergillus* section *Nigri*.

### Plug extraction

For standard screening three plugs were taken from one colony, by use of a 6 mm steel plug drill, close to the center of the colony, beside the raisin or other fruit, if added. If any sclerotia could be observed, the agar plugs were taken to include the sclerotia. For comparison between sclerotial extracts and agar colony extracts five plugs were taken from one colony, covering the diameter of the colony (only when HPLC-MS analysis was intended). The plugs were transferred to 2 mL vials and 750 µL methanol: dichloromethane: ethyl acetate (1∶2∶3 v/v/v) containing 1% formic acid was added. For HPLC-MS analysis each sample was added 40 µL chloramphenicol in ethanol (500 µg/mL) as an internal standard. The vials were placed in an ultrasonic bath (Branson 3210) for 60 minutes. The extracts were then transferred to clean vials and evaporated to dryness. This was either achieved by leaving the vials in a fume hood over night or by applying nitrogen airflow at 25–32°C. After evaporation, 500 µL MeOH was added and the samples were then ultrasonicated for 20 minutes to re-dissolve the fungal metabolites. The extracts were then filtered using a 0.45 µm polytetrafluoroethene (PTFE) filter into clean vials [51].

### Sclerotium extraction

The fungi were three-point inoculated on solid CYA plates with biotin and raisins and incubated at 25°C for 7 days in the dark. Sclerotia were harvested by applying pure H_2_O to the plate and carefully harvested with a Drigalski spatula. The liquid including the sclerotia and spores was filtered through sterile Miracloth and washed with pure water and ethanol. This allowed for spores to pass through, while sclerotia stayed in the filter. Sclerotia were transferred to Eppendorf tubes and washed with water to remove remaining spores. After evaporation of excess H_2_O, two big (diam. 4 mm) and two small steel balls (diam. 2 mm) were added to each tube. The tubes were cooled with liquid nitrogen and shaken using a frequency of 2000/min for 2×60 seconds in a sonic Micro Dismembrator U homogenizer (Sartorius). The tubes were then heated to 30°C and shaken again for 2×60 second. To each tube 40 µL chloramphenicol and 0.750 mL methanol: dichlormethane: ethyl acetate (1∶2∶3 v/v/v) containing 1% formic acid was added and the extracts were transferred to clean 2 mL vials. The samples were ultrasonicated (Branson 3210 sonicator) for 1 h and the extracts were then transferred to clean vials and the solvent was evaporated. The samples were re-dissolved in 500 µL MeOH, ultrasonicated for 20 minutes and filtered into fresh vials using 0.45 µm PTFE filters.

### UHPLC-DAD analysis

The analyses of the 3 plug extracts were performed using Ultra High Performance Liquid Chromatography-diode array detection (UHPLC-DAD) as described by Frisvad and Thrane [52] as modified by Houbraken et al. [53]. UV spectra were recorded from 190 to 700 nm. For florescence detection, the excitation wavelength was 230 nm and the emission wavelength was 333 nm. This allows for a sensitive detection of extrolites with an indole chromophore, including aflavinins.

### UHPLC-DAD-HRMS analysis

The analyses were performed using a Ultra High Performance Liquid Chromatograpy-UV/Vis-High Resolution Mass Spectrometer (UHPLC-DAD-HRMS) on a maXis G3 oa Qq-TOF mass spectrometer (Bruker Daltonics, Bremen, Germany) equipped with an electrospray (ESI) source and connected to an Ultimate 3000 UHPLC system (Dionex, Sunnyvale, CA). The column used was a reverse-phase Kinetex 2.6 µm C_18_, 100×2.1 mm (Phenomenex, Torrance, CA) and the column temperature was maintained at 40°C. A linear water-acetonitrile gradient was used (both buffered with 20 mM formic acid) starting from 10% acetonitrile and increased to 100% in 10 minutes and maintaining this for 3 minutes before returning to the starting conditions in 0.1 minute and staying there for 2.4 minutes before the following run. A flow rate of 0.4 mL min^−1^ was used. HRMS was performed in ESI+ with a data acquisition range of 10 scans per second at *m/z* 100–1000. The MS was calibrated by use of the internal standard sodium formate, which was automatically infused prior to each run. UV spectra were collected at wavelengths from 200–700 nm. Data processing was performed using the Bruker software DataAnalysis and TargetAnalysis.

## Results

### Aspergillus niger and sclerotia

**Figure 1:**
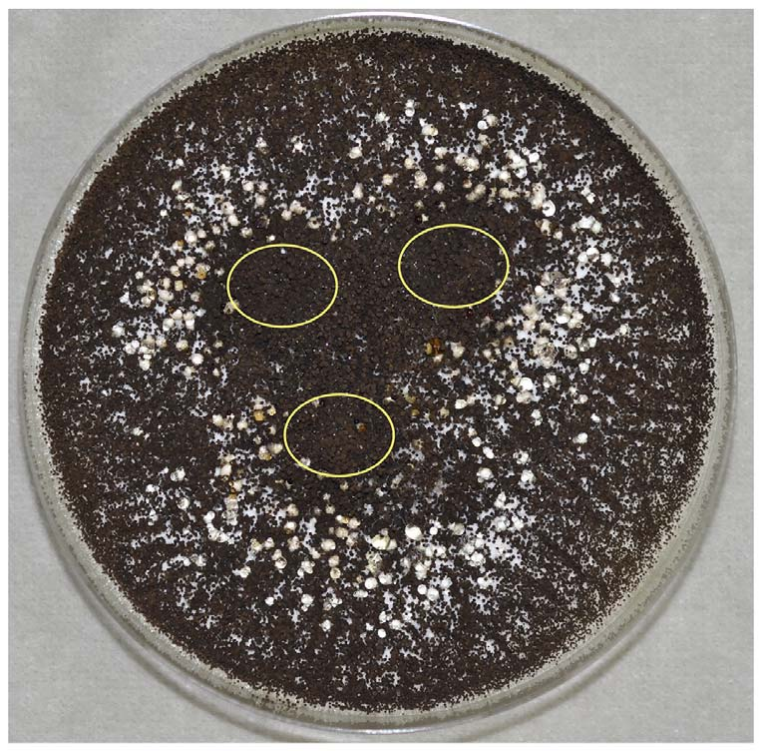
Numerous cream-coloured sclerotia produced on CYAR agar can be seen surrounding the raisins and the usual heavy sporulation caused by black *Aspergillus niger* IBT 29019 heads. The ellipses added show the position of the raisins, which are covered with black aspergilla.

**Figure 2:**
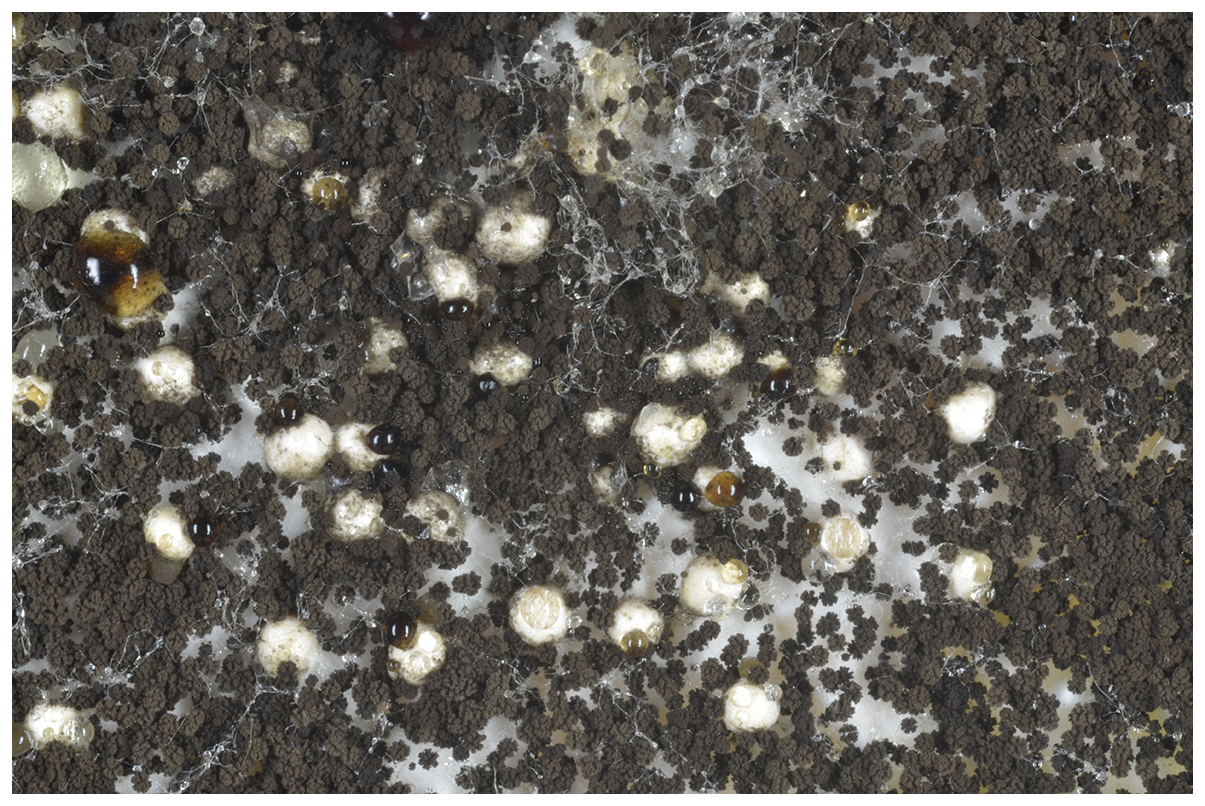
Approximately 41 white sclerotia can be observed on a close-up of *Aspergillus niger* IBT 29019 growing on Czapek yeast autolysate agar with raisins. Note the clear or brown exudate droplets associated with most sclerotia.

No strains of *Aspergillus niger sensu stricto*, tested in this study or in our earlier studies, produced sclerotia on any of the conventional media tested. The closely related sibling phylospecies *A. welwitchiae* [54], formerly called *A. awamori* [54]–[55] also did not produce sclerotia on any medium. Initially *A. niger* IBT 29019 and IBT 24631 produced sclerotia on a medium made with dark Californian raisins added to the medium. Interestingly sclerotia were only formed on CYA medium with whole raisins, and not on the same medium with macerated raisins. It was decided to add three whole sterilized pieces of raisin on top of the CYA medium (CYAR), and the experiments were repeated with several brands of raisins from California, Turkey, Chile and Argentina (Table S1 in File S1). IBT 29019 and IBT 24631 produced sclerotia surrounding all organic and conventional dark raisins tested, but not white raisins (the latter had been treated with sulfur dioxide), and did not produce sclerotia on the control CYA agar medium. The sclerotia formed in *A. niger* (Fig. 1 and 2) were similar to those produced by *Aspergillus carbonarius* and *A. tubingensis*, i.e. large cream-coloured sclerotia, which were globose to ovoid and 500–700 µm in diameter. The strain *A. niger* IBT 24634 did not produce sclerotia under any conditions. The experiments using CYA agar with raisins were repeated several times and in nearly all cases sclerotia were formed. In few experiments sclerotia were not produced on CYA agar with whole raisins by IBT 29019, and in those cases sclerotium production could then be induced by keeping the conidia used for inoculation (conidia on agar plugs from a colony) of the media in a freezer at −18°C for at least three weeks. When this freezing step was done, sclerotium production was entirely consistent in IBT 29109 and IBT 24631.

A set of three isolates of *Aspergillus niger* (IBT 29019, IBT 24631 and IBT 24634) were first tested on the medium CYA and CYA with different pieces of whole fruits added or inoculated on whole rice (Table 1). Certain fruits stimulated sclerotium production in IBT 29019, including raisin, mulberry, blueberry, cranberry, apricot, prune, and mango peel, all placed on CYA agar, but also on 40% mango macerate in agar, and 50% white rice or 50% brown rice (rice media without added agar). Sclerotia were not formed on media with goji berries, green coffee beans, red kidney beans, black pepper, 4% or 40% papaya macerate in agar, 4% mango macerate in agar or on any of the media CYA, malt extract agar (MEA), yeast extract sucrose (YES) agar, oatmeal agar (OAT), potato dextrose agar (PDA), or Wickerhams antibiotic test medium (WATM). However regarding the latter 5 media, conidia were not frozen before inoculation, so it cannot be excluded that these media may be inducing sclerotium production if inoculated after a freeze treatment. The strain *A. niger* IBT 24631 only produced sclerotia on some of the media: CYA agar with raisins (CYAR), in some cases on CYA with prune or apricot, and on whole white or brown rice (50%). Furthermore in one case sclerotium production was seen on whole corn (50%, no agar added) in *A. niger* IBT 24631 but not in IBT 29019. Neither strain produced sclerotia on whole unpolished rice, polished rice, dried whole corn or dried kidney beans, except for the single incidences mentioned above. If produced, sclerotia were observable after 7 days of incubation, and only rarely were additional sclerotia produced after 7 days. All sclerotia produced were cream-white first, and darkening becoming beige to steel grey in age.

### Strains of *Aspergillus niger* producing sclerotia and sclerotial indoloterpenes

**Figure 3:**
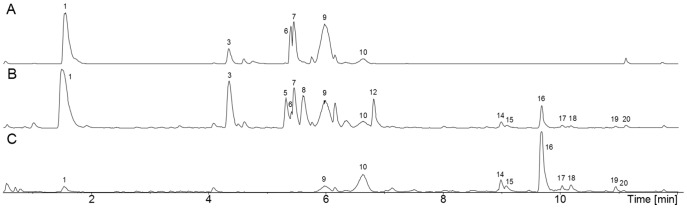
Ultra high performance liquid chromatography time of flight high resolution mass spectrometry electrospray ionization + base peak (UHPLC-TOF-HRMS ESI+ BP) chromatogram of *Aspergillus niger* (IBT 29019) extracts. **A**: Plug extraction from growing and sporulating culture with no sclerotium production (CYA agar with biotin). **B**: Plug extraction from growth with sclerotium production (CYAR with biotin). **C**: Sclerotium extraction (from CYAR with biotin). 1) Nigragillin, 2) Pyranonigrin A, 3) Fonsecin, 4) Aurasperone/nigerasperone analog, 7) Tensidol B, 11) Kotanin, 12) Flavasperone analog, 13) Rubrofusarin B, 14) Aflavinine analog, 15) Aflavinine analog, 17) Anominine, 18) 10,23-dihydro-24,25-dehydroaflavinine.

In all cases the presence of sclerotia in *Aspergillus niger* was confirmed chemically by the presence of sclerotium associated aflavinin-type apolar indoloterpenes. Consistently, indoloterpenes of the 10,23-dihydro-24,25-dehydroaflavinine type were produced in all sclerotium containing media, but indoloterpenes were not produced in any cases on media where sclerotia were not present (Table 1). Two major indoloterpenes were produced by the strains of *A. niger* IBT 29019, and one major indoloterpene was produced by *A. niger* IBT 24631 as determined by both diode array and fluorescence detection. The total number of indoloterpenes ranged from one to 13, with highest numbers of indoloterpernes produced on brown rice (Table 2). Most of the indoloterpenes produced had an ordinary indol chromophore, but there were also apolar sclerotial metabolites with UV spectra not seen earlier, indicating that some of these had a somewhat modified chromophore but still quite similar to the normal indol chromophore. *Aspergillus niger* IBT 29019 produced 10,23-dihydro-24,25-dehydroaflavinine and two additional aflavinines, in addition to a fourth indoloterpene, which had the same formula (C_28_H_39_NO), UV spectrum and retention time as anominine (Fig. 3). On brown rice IBT 29019 produced 13 different apolar sclerotial metabolites, but most of them are as yet not structure elucidated. Anominine was formerly known as nominine [56].

Ten recently isolated strains isolated from Californian raisins produced sclerotia on CYAR, including IBT 29019 = CBS 133818, IBT 28998, IBT 28999, IBT 29000, IBT 29001, IBT 29003, IBT 29005, IBT 29006, IBT 29007 and IBT 29020 (Table 1), whereas four strains from the same raisin sample did not (IBT 29002, IBT 29004, IBT 29021, and IBT 29023) (Table S2 in File S1). One strain from black pepper, IBT 24631 = CBS 133816 could produce sclerotia on CYAR, while another strain from the same pepper sample, IBT 24634 = CBS 133817, could not. A number of culture collection strains of *A. niger* were also tested on CYAR and only 1 strain, NRRL 599, previously reported to produce high levels of citric acid formed sclerotia on CYAR, while other industrial and genome sequenced strains CBS 513.88 and NRRL 328, the culture ex type of *A. niger* NRRL 326 and other strains: CBS 139.52, CBS 119725, NRRL 363, NRRL 593, NRRL 611, NRRL 612, NRRL 2372, NRRL 3112, and NRRL 4757 did not produce sclerotia on CYAR (Table S2 in File S1).

14 strains of the sibling species of *A. niger*, *A. welwitchiae* were also tested on CYAR, and did not produce sclerotia: CBS 102.12, CBS 618.78, IBT 29098, ITEM 7097, NRRL 320, NRRL 340, NRRL 362, NRRL 372, NRRL 567, NRRL 595, NRRL 604, NRRL 2001, NRRL 4851, and NRRL 6408 (Table S2 in File S1).

All 12 *A. niger* strains producing sclerotia also produced 1–13 detectable aflavinins or related apolar indoloterpenes, including 10,23-dihydro-24,25-dehydroaflavinine (Table 2), while none of the non-sclerotium producing strains produced any aflavinin-like molecules.

In *A. niger* IBT 29019, sporulating cultures contained known *A. niger* extrolites including nigragillin, pyranonigrin A, tensidol B, kotanin and naphtho-γ-pyrones. However in the sclerotia alone, only aflavinin molecules and an indoloterpene tentatively identified as anominine were produced, with only traces of nigragillin (Fig. 3).

### Strains of *Aspergillus* series *Nigri* producing sclerotia and sclerotial indoloterpenes

**Figure 4:**
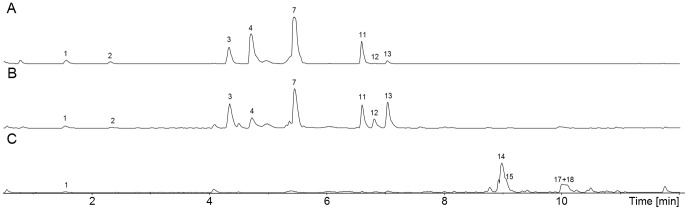
Ultra high performance liquid chromatography time of flight high resolution mass spectrometry electrospray ionization + base peak (UHPLC-TOF-HRMS ESI+ BP) chromatogram of *Aspergillus tubingensis* (IBT 16833 = CBS 161.79 = NRRL 4700) extracts. **A**: Plug extraction from colony area with no sclerotia (CYAR with biotin). **B**: Plug extraction from colony area with sclerotia (CYAR with biotin). **C**: Sclerotium extraction (from CYAR with biotin). 1) Nigragillin, 3) Fonsecin, 5) TMC-256A1, 6) Asperazine, 7) Tensidol B, 8) Fonsecin B, 9) Aurasperone C, 10) Aurasperone B, 12) Flavasperone analog, 14) Aflavinine analog, 15) Aflavinine analog, 16) Aflavinine analog, 17) Anominine, 18) 10,23-dihydro-24,25-dehydroaflavinine, 19) Aflavinine analog, 20) Aflavinine analog. From this strain TePaske et al. [20], [21], [22] isolated 14-epi-14-hydroxy-10,23-dihydro-24,25-dehydroaflavinine, 10,23-dihydro-24,25-dehydro-21-oxo-aflavinine, 10,23-dihydro-24,25-dehydroaflavinine, tubingensin A and B, and dehydrotubingensin A and B.

In *Aspergillus* series *Nigri* [44], four species did not produce sclerotia or indoloterpenes under any conditions: *A. eucalypticola*, *A. lacticoffeatus*, *A. vadensis* and *A. welwitchiae*. On the other hand *A. costaricaensis* and *A. piperis* produced sclerotia on all media at the expense of black conidiophores heads and sclerotial metabolites included 10,23-dihydro-24,25-dehydroaflavinine and 12 to 13 other apolar indoloterpenes. *A costaricaensis* also produced aspernomine [57]. Some strains of *A. tubingensis* (NRRL 4700) and *A. luchuensis* (IBT 24821, IBT 24825) produced sclerotia readily on CYA agar, while other strains of these two species needed raisins to induce sclerotium formation. The sclerotia of *A. luchuensis* formed readily on CYA tended to aggregate, they were cream coloured and very large, 800–1300 µm in diameter. The sclerotia of *A. costaricaensis* were also very large, from 1200–1800 µm, and those of *A. piperis* were yellowish to pinkish brown, 1000–1700 µm in diameter [5]. The sclerotia in *A. tubingensis* were the smallest in this series, 500–800 µm, first cream-coloured, later becoming pinkish to light brown. While sclerotium producing *A. luchuensis* produced 10,23-dihydro-24,25-dehydroaflavinine and 3 other apolar indoloterpenes, sclerotial *A. tubingensis* strains produced 10,23-dihydro-24,25-dehydroaflavinine, aspernomine and up to 19 additional apolar indoloterpenes on CYAR. We did not detect tubingensin A and B [21]–[22] and dehydrotubingensin A and B [23] on CYAR, but we have formerly identified these in NRRL 4700 cultures on the media CYA and YES agar. Furthermore 14-epi-14-hydroxy-10,23-dihydro-24,25-dehydroaflavinine and 10,23-dihydro-24,25-dehydro-21-oxo-aflavinine, earlier detected in *A. tubingensis* NRRL 4700 [22] were not detected on CYAR. This indicates that at least 26 different sclerotial indoloterpenes can be produced by *A. tubingensis*. In the conidial areas of *A. tubingensis* IBT 16833 ( = NRRL 4700) we detected nigragillin, asperazine, and naphtho-γ-pyrones (Fig. 4). In the sclerotia, and sclerotial parts of the agar cultures (CYAR) we detected 6 aflavinins by UHPLC-TOF-HRMS and UHPLC-DAD and a compound tentatively identified as anominine. These indoloterpenes were not found in cultures with conidial heads only. It appears that the indoloterpenes and the naphtho-γ-pyrone, aurasperone B, are up-regulated in sclerotial cultures (Fig. 4).

Sclerotia were detected for the first time in *A. brasiliensis* and *A. neoniger*. *A. brasiliensis* IBT 28177 produced two detectable sclerotial indoloterpenes, while *A. neoniger* IBT 30603 and IBT 20973 produced 12 sclerotial apolar indoloterpenes on CYAR, including 10,23-dihydro-24,25-dehydroaflavinine and aspernomine (Table 2). Aspernomine was tentatively identified for the first time in *A. tubingensis, A. neoniger* and *A. costaricaensis*, based on comparison with aspernomine in extracts of *Aspergillus nomius* and an identical characteristic UV spectrum and retention time in all extracts of these fungi.

### Strains of *Aspergillus* series *Carbonaria* producing sclerotia and sclerotial indoloterpenes

In *Aspergillus* series *Carbonaria* two species, *A. sclerotiicarbonarius* and *A. sclerotioniger* produce sclerotia readily on most media. The sclerotia of *A. sclerotioniger* were 1000–1600 µm in diameter, and were yellow to red brown [5], while those of *A. sclerotiicarbonarius* were of the same colours as *A. sclerotioniger* and 600–1600 µm in diameter [7]. The sclerotia of *A. carbonarius* were cream coloured to light brown to pinkish to greyish brown in age, with a diameter of 800–1300 µm [1]. The sclerotia of *A. ibericus* were cream to light brown, and smaller than those of *A. carbonarius*: 700–1000 µm in diameter. *A. sclerotioniger* produces ochratoxin A, but not indoloterpenes on CYA or CYAR. *A. sclerotiicarbonarius* produced three indoloterpenes with a paspalinine chromophore, but none of these have been structure elucidated. Like *A. sclerotioniger*, strains of *A. carbonarius* produced ochratoxin A in the sclerotia and no indoloterpenes were detected (Table 2). While non-sclerotial strains of *A. carbonarius* can produce ochratoxin A, sclerotium production increases the ochratoxin A titre in this species [19]. In *A. ibericus* CBS 121593, CYAR induced production of sclerotia and production of one indoloterpene in those sclerotia. As is the case for *A. lacticoffeatus* and *A. niger* in series *Nigri*, no isolate has been found that produce both ochratoxin A and apolar indoloterpenes in series *Carbonaria*. This is the first report on sclerotium production and presence of an aflavinine in *A. ibericus* (Table 2).

### Strains of *Aspergillus* series *Heteromorpha* producing sclerotia and sclerotial indoloterpenes

*Aspergillus ellipticus* and *A. heteromorphus* produced sclerotia when grown on CYAR. Both species have earlier been reported to produce sclerotia. A sclerotium producing sector of *A. ellipticus* was separated and named as a new species, *A. helicothrix*, by Al-Musallam [3]. Later research has shown that *A. ellipticus* and *A. helicothrix* are the same species [6]. We could induce sclerotium formation in *A. ellipticus* on CYAR, but we did not detect any sclerotium associated extrolites in that species. *A. heteromorphus* was originally claimed to produce sclerotia [10], but Al-Musallam [3] and Samson et al. [6] could not confirm this. We observed sclerotium production in *A. heteromorphus* CBS 101889 on CYAR. In this single case the sclerotia were only produced on the raisins and the sclerotia were the smallest observed in this study (300–500 µm) and becoming darker (steel grey) in age than those of the other species in *Aspergillus* section *Nigri*. We did not detect any sclerotium associated extrolites in this species, however (Table 2).

### Strains of *Aspergillus* series *Homomorpha* producing sclerotia and sclerotial indoloterpenes

We did not detect any sclerotia in *A. homomorphus*, but we did observe sclerotia in *A. saccharolyticus* when isolates of this species (IBT 30881 and IBT 28231) grew on CYAR. The sclerotia of *A. saccharolyticus* were small, 300–500 µm in diameter and white to cream coloured. One apolar indoloterpene with a paspalinine chromophore was detected in *A. saccharolyticus* (Table 2). This is the first report on sclerotium production by *A. saccharolyticus*, as sclerotia were not reported in the original description of this species [58].

### Strains of *Aspergillus* series *Aculeata* producing sclerotia and sclerotial indoloterpenes

We did not detect sclerotia in *A. indologenus* or *A. uvarum* when isolates of these species were grown on CYAR. *A. indologenus* did produce extrolites that indicate the possibility that this species may eventually produce sclerotia given the right culturing conditions as reported by Al-Musallam [3], i.e. okaramins, also produced by sclerotial *A. aculeatus* and mid-polar indol-alkaloids, in common with sclerotial *A. violaceofuscus*. Strains of three species occasionally produced sclerotia on CYA agar, but produced them regularly on CYAR: *A. aculeatinus*, *A. aculeatus* and *A. brunneoviolaceus*/*A. fijiensis* and *A. japonicus*. The sclerotia of all the species in this group were small (300–500 µm) and white to cream coloured. All species produced apolar indoloterpenes of the aflavinine or paspalinine type, but they produced different profiles of apolar indoloterpenes. The only known aflavinine identified was 10,23-dihydro-24,25-dehydroaflavinine in *A. aculeatus* and *A. japonicus*. *A. aculeatus* also produced one apolar indoloterpene of the paspalinine type and okaramins. *A. violaceofuscus* produced sclerotia, but only mid-polar indol-alkaloids were detected. *A. floridensis* and *A. trinidadensis* also produced sclerotia on CYAR. *A. floridensis* produced 10,23-dihydro-24,25-dehydroaflavinine and *A. trinidadensis* produced an apolar indoloterpene with a paspalinine chromophore (Table 2). The *extype* strain of *Saitoa japonica* (IMI 240698) [2] produced abundant sclerotia on all media, and was allocated to *Aspergillus aculeatus* based on chemotaxonomic evidence, however *S. japonica* IMI 240698 also has many similarities with *Aspergillus brunneoviolaceus*. This strain should be sequenced to allow a final identification. IMI 240698 produced three okaramins, one calbistrin, but in contrast to *A. brunneoviolaceus* it did not produce aspergillimide. After two month of incubation of *Saitoa japonica* IMI 240698 on MEA at room temperature the sclerotia were examined for ascocoma production, but these were not observed. This is the first report of sclerotium production in *A. floridensis* and *A. trinidadensis*, not reported in the original description of the two species [9].

## Discussion

*Aspergillus niger* is one of the most important industrial microorganisms used, but is also a common contaminant of foods, so it is of high importance whether this organism can recombine or whether it is a clonal fungus. Sclerotia are necessary structures if a fungus has to produce a perfect state, at least in *Aspergillus* sections *Flavi*, *Nigri* and *Circumdati* which will produce one to several ascomata within a sclerotium [25]–[27], [31]–[32], [59], either homothallically or heterothallically. Even though mating-type genes have been found in *A. niger* [16], [18], the absence of sclerotia in strains of *A. niger* has precluded the discovery of a perfect state in this species. We show here that addition of whole raisins or other plant parts to the medium CYA can induce sclerotium production in certain strains of *A. niger*.

Since indoloditerpenes are exclusively found in the sclerotia of *Aspergillus* sections *Nigri*, *Flavi* and *Circumdati* and since these indoloterpenes have been reported to be antiinsectan extrolites [19]–[23], [57], [60] they appear to be produced to protect sclerotia from being eaten by insects. As can be seen from the results obtained here, the sclerotia may contain a high number of indoloterpenes, many of which have been shown to be antiinsectan [60]. Sclerotium formation seems to be lost in *Aspergillus* strains cultured for a long time, but may also be lost in nature because these fungi are ever more depending on asexual conidial survival in domesticated landscapes [19], [25]. Ascospore formation inside hard sclerotia in *Aspergillus* subgenus *Circumdati* is a very slow process, often taking from 3–12 month, and apparently requiring special physical and chemical factors to be initiated, irrespective of homothallism or heterothallism [2], [26]–[29], [31]–[33], [59], and so the sexual potential of these fungi appears to be weak. This is also seen in this study: often it is only a minority of strains in the ubiquitous species *A. niger* and *A. tubingensis* that can produce sclerotia, even after stimulation by plants parts such as seeds and fruits.

It was observed that no isolates of *A. niger* producing ochratoxin A could produce sclerotia or indoloterpenes, but more OTA producing isolates need to be examined before a conclusion on this interesting phenomenon can be made.

The first observation of a perfect state in any species of *Aspergillus* section *Nigri* was reported by Rajendran and Muthappa in 1980 [2]. In this case ascomata were found after 3 month of growth on malt extract agar. Since this fungus is homothallic, no mating partners needs to be used. It should be examined whether other sclerotium producing strains in the uniseriate black aspergilla are also homothallic. Twenty three years later Darbyshir et al. [31] succeeded to induce a perfect state in *A. sclerotiicarbonarius* and Horn et al. [32] one in *A. tubingensis*, and they used opposite mating types of naturally sclerotium producing isolates and plated these on a mixed cereal agar. Our experience has shown that cereal based media are not the most effective for sclerotium formation in species in *Aspergillus* section *Nigri*, but that the medium CYA with whole black raisins could be an ideal medium for crossing of opposite mating types of *A. niger*. It is important to note the freezing of the conidia for 3 weeks before inoculation was often necessary to induce sclerotium production on CYAR. In the initial experiments freezing was not used, and we still observed sclerotium production in *A. niger* IBT 29019. We suspect these original observations of sclerotia in *A. niger* were based on the fact that the cultures came directly from silica gel, because after several conidial transfers *A. niger* IBT 29019 failed to produce sclerotia on CYAR. The ability to produce sclerotia was restored when conidia were frozen before inoculation, so we used this treatment when testing other species for sclerotium production on CYAR. Even though sclerotium production has been reported from *A. niger* earlier [1],[3],[61],[62], the strains reported to produce them were actually later shown to be *A. costaricaensis*, *A. tubingensis* or *A. carbonarius* [5], [6], [8]. Other fruits than raisins, such as blueberries, cranberries, mulberries, apricot, prune, mango were also useable for inducing sclerotium formation by *A. niger* IBT 29019 on CYA agar, while goji berries, white raisin preserved with sulfur dioxide, papaya, kidney beans, black pepper and green coffee beans were not. Whole white or brown rice also was effective for inducing sclerotium and indoloterpene production. Kjer et al. [63] recommended using whole rice in the screening for secondary metabolites in fungi, and whole rice may be a valuable addition for screening purposes to known effective media for secondary metabolite production, such as CYA agar and YES agar [64]. However, most of our experiments were done with the CYAR medium. When tested on several types and brands of black raisins, they all induced sclerotium production on CYA agar. However, macerated raisins did not induce sclerotium production, so it appears that the skin or waxy skin coating of the raisins may be the crucial factor. In grape wax oleanolic acid and octacosanoic acid are predominant [65], while in wheat leaves the wax 1-octacosanol is dominant [66] so it is not unlikely that such wax compounds, when present in high concentrations as on the surface of a fruit, are influencing sclerotium production. We tried to add sunflower oil to CYA, as this oil is often used for coating raisins to give a shiny surface, but on such a medium no sclerotia were formed. It remains to be seen what the sclerotium inducing factors are, but they appear to be based on a complicated mechanism, as some species in *Aspergillus* section *Nigri* produce sclerotia readily, while others only produce them under very specific conditions, even within the same species. Wicklow et al. [24] used whole corn kernels for inducing sclerotium formation in *A. carbonarius*, and concerning *A. niger* IBT 24631, few sclerotia were produced on whole corn kernels, but in general raisins on CYA agar is a more effective alternative. Raper and Fennell [1] induced sclerotium formation in *A. carbonarius* WB 346 by using steep agar, which is Czapek agar with 5 g/l corn steep liquor, so it appears that corn ingredients has a certain positive effect on sclerotium formation. Batista and Maia [10] also reported sclerotium production by *A. heteromorphus* on Czapek agar with 1% corn steep liquor. We tried another corn steep liquor containing medium, WATM, for inducing sclerotium formation in *A. niger* and *A. tubingensis*, but this medium did not work as well as in *Aspergillus* section *Flavi*, where it induces sclerotium formation effectively [67].

## Conclusion

Adding whole autoclaved fruits or grains to an agar medium stimulates sclerotium production in some strains of many species of the black Aspergilli, notably *A. niger*, but also *A. brasiliensis*, *A. heteromorphus, A. ibericus, A. luchuensis*, *A. neoniger*, and the uniseriate black Aspergilli *A. aculeatinus, A. aculeatus, A. brunneoviolaceus/A. fijiensis, A. floridensis, A. japonicus, A. saccharolyticus, A. trinidadensis* and *A.violaceofuscus*. Pre-freezing of the conidium inoculum enhanced sclerotium production. Raisins also stimulated sclerotium production further in species that often produced them readily, such as some strains of *A. tubingensis* and *A. carbonarius*, and all strains of *A. costaricaensis*, *A. piperis*, *A. sclerotiicarbonarius* and *A. sclerotioniger* leaving *A. eucalypticola*, *A. lacticoffeatus*, *A. uvarum*, *A. vadensis* and *A. welwitchiae* the only species in *Aspergillus* section *Nigri* where sclerotia have not yet been detected. 10,23-dihydro-24,25-dehydroaflavinine-type apolar indoloterpenes were produced by most sclerotial species, but species related to *A. carbonarius* preferentially produced ochratoxin A in the sclerotia. The aflavinin and other apolar indoloterpene type metabolites were produced in the sclerotia only.

## Supporting Information

File S1 Table S1. Table S1 is a list of fruits or seeds used to induce sclerotium production in *Aspergillus niger* strains. Table S2. Table S2 is a list of isolates in *Aspergillus brasiliensis*, *A. niger* and *A. welwitchiae* that do not produce sclerotia, even when stimulated by whole raisins in CYA agar in addition to a pre-freezing step.
(DOCX)

## References

[1] Raper KB, Fennell DI (1965) The genus *Aspergillus*. Baltimore, MD, USA: Williams and Wilkins. 686 p.

[2] Rajendran C, Muthappa BN (1980) *Saitoa*, a new genus of Plectomycetes. Proc Indian Acad Sci (Plant Sci) 89: 185–191.

[3] Al-Musallam A (1980) Revision of the black *Aspergillus* species. Baarn: Centraalbureau voor Schimmelcultures. 92 p.

[4] Jarvis WR, Traquair JA (1985) Sclerotia of *Aspergillus aculeatus* . Canad J Bot 63: 1567–1571.

[5] Samson RA, Houbraken JAMP, Kuijpers AFA, Frank JM, Frisvad JC (2004) New ochratoxin or sclerotium producing species in *Aspergillus* section *Nigri* . Stud Mycol 50: 45–61.

[6] Samson RA, Noonim P, Meijer M, Houbraken J, Frisvad JC, et al (2007) Diagnostic tools to identify black Aspergilli. Stud Mycol 59: 129–145. 18490945 10.3114/sim.2007.59.13 PMC2275192

[7] Noonim P, Mahakarnchanakul W, Varga J, Frisvad JC, Samson RA (2008) Two novel species of *Aspergillus* section *Nigri* from Thai coffee beans. Int J Syst Evol Microbiol 58: 1727–1734. 18599725 10.1099/ijs.0.65694-0

[8] Varga J, Frisvad JC, Kocsubé S, Brankovics B, Tóth B, et al (2011) New and revisited species in *Aspergillus* section *Nigri* . Stud Mycol 69: 1–17. 21892239 10.3114/sim.2011.69.01 PMC3161757

[9] Jurjević Ž, Peterson SW, Stea G, Solfrizzo M, Varga J, Hubka V, Perrone G (2012) Two novel species of *Aspergillus* section *Nigri* from indoor air. IMA Fungus 3: 159–173. 23355969 10.5598/imafungus.2012.03.02.08 PMC3539319

[10] Batista AC, Maia H da Silva (1957) Alguns *Aspergillus* contaminantes de culturas. An Soc Biol Pernamb 15: 181–237.

[11] Porter CL, Coats JH (1957) Protoplasmic connections between cells in sclerotia of certain *Aspergillus* and *Penicillium* species. Mycologia 49: 895–896.

[12] Coats JH (1959) Physiological studies concerning the formation of sclerotia in *Aspergillus niger* . Diss Abstr 20: 1544–1545.

[13] Rai JN, Tewari JP, Sinha AK (1967) Effect of environmental conditions on sclerotia and cleistothecia production in *Aspergillus* . Mycopathol Mycol Appl 31: 209–224. 6031297 10.1007/BF02053418

[14] Frisvad JC, Larsen TO, Thrane U, Meijer M, Varga J, et al (2011) Fumonisin and ochratoxin production in industrial *Aspergillus niger* strains. PLOS ONE 6: e23496. 21853139 10.1371/journal.pone.0023496 PMC3154942

[15] Peters I, Rippel-Baldes A (1949) Über das Vorkommen verschiedener Rassen von *Aspergillus niger* van Tiegh. im Boden. Arch Microbiol 14: 203–211.

[16] Pel HJ de Winde JH, Archer DB, Dyer PS, Hofmann G, et al (2007) Genome sequencing and analysis of the versatile cell factory *Aspergillus niger* CBS 513.88. Nat Biotechnol 25: 221–231. 17259976 10.1038/nbt1282

[17] Baker S (2006) *Aspergillus niger* genomics: Past, present and into the future. Med Mycol 44 Suppl 1S17–S21. 17050415 10.1080/13693780600921037

[18] Andersen MR, Salazar MP, Schaap PJ, van de Vondervoort, PJI, Culley D, et al (2011) Comparative genomics of citric-acid producing *Aspergillus niger* ATCC 1015 versus enzyme-producing CBS 513.88. Genome Res 21: 885–897. 21543515 10.1101/gr.112169.110 PMC3106321

[19] Wicklow DT (1988) Metabolites in the coevolution of fungal chemical defence systems. In: Pirozynski KA & Hawksworth DL (eds) Coevolution of fungi with plants and animals. Academic Press, London, pp. 173–201.

[20] TePaske MR, Gloer JB, Wicklow DT, Dowd PF (1989a) Tubingensin A: An antiviral carbazole alkaloid from the sclerotia of *Aspergillus tubingensis* . J Org Chem 54: 4743–4746.

[21] TePaske MR, Gloer JB, Wicklow DT, Dowd PF (1989b) Tubingensin B: A cytotoxic carbazole alkaloid from the sclerotia of *Aspergillus tubingensis* . Tetrahedron Lett 30: 5965–5968.

[22] TePaske MR, Gloer JB, Wicklow DT, Dowd PF (1989c) Three new aflavinines from the sclerotia of *Aspergillus tubingensis* . Tetrahedron 45: 4961–4968.

[23] Sings HL, Harris GH, Dombrowski AEW (2001) Dihydrocarbazole alkaloids from *Aspergillus tubingensis* . J Nat Prod 64: 836–838. 11421760 10.1021/np000613p

[24] Wicklow DT, Dowd PF, Alfatafta AA, Gloer JB (1996) Ochratoxin A: An antiinsectan metabolite from the sclerotia of *Aspergillus carbonarius* NRRL 369. Can J Bot 42: 1100–1103. 10.1139/m96-141 8941986

[25] Dyer PS, O'Gorman CM (2012) Sexual development and cryptic sexuality in fungi: insights from *Aspergillus* species. FEMS Microbiol Rev 36: 165–192. 22091779 10.1111/j.1574-6976.2011.00308.x

[26] Horn BW, Moore GG, Carbone I (2009a) Sexual reproduction in *Aspergillus flavus* . Mycologia 101: 423–429. 19537215 10.3852/09-011

[27] Horn BW, Moore GG, Carbone I (2009b) The sexual state of *Aspergillus parasiticus* . Mycologia 101: 275–280. 19397202 10.3852/08-205

[28] Horn BW, Moore GG, Carbone I (2011) Sexual reproduction in aflatoxin-producing *Aspergillus nomius* . Mycologia 103: 174–183. 20943531 10.3852/10-115

[29] Udagawa S, Uchiyama S, Kamiya S (1994) *Petromyces muricatus*, a new species with an *Aspergillus* anamorph. Mycotaxon 52: 207–214.

[30] Frisvad JC, Samson RA (2000) *Neopetromyces* gen. nov. and an overview of teleomorphs of *Aspergillus* subg. *Circumdati* . Stud Mycol 45: 201–207.

[31] Darbyshir HL, van de Vondervoort PJI, Dyer PS (2013) Discovery of sexual reproduction in the black Aspergilli. Fung Gen Rep (Suppl) 60: 290 (abstract # 687).

[32] Horn BW, Olarte RA, Peterson SW, Carbone I (2013) Sexual reproduction in *Aspergillus tubingensis* from section *Nigri* . Mycologia 105: 1153–1163. 23709489 10.3852/13-101

[33] Kück U, Böhm J (2013) Mating type genes and cryptic sexuality as tools for genetically manipulating industrial molds. Appl Microbiol Biotechnol 97: 9609–9620. 24085397 10.1007/s00253-013-5268-0

[34] Nielsen KF, Mogensen JM, Johansen M, Larsen TO, Frisvad JC (2009) Review of secondary metabolites and mycotoxins from the *Aspergillus niger* group. Anal Bioanal Chem 395: 1225–1246. 19756540 10.1007/s00216-009-3081-5

[35] Mogensen JM, Nielsen KF, Frisvad JC, Samson RA Thrane U (2009) Effect of temperature and water activity on the production of fumonisin B_2_ by *Aspergillus niger* and *Fusarium* species. BMC Microbiol 9: 281. 20043849 10.1186/1471-2180-9-281 PMC2811119

[36] Mogensen JM, Frisvad JC, Thrane U, Nielsen KF (2010) Production of fumonisin B_2_ and B_4_ by *Aspergillus niger* on grapes and raisins. J Agric Food Chem 58: 954–958. 20014861 10.1021/jf903116q

[37] Noonim P, Mahakarnchanakul W, Frisvad JC, Samson RA (2008) Distribution of ochratoxin A-producing fungi in coffee beans (*Coffea arabica* and *Coffea robusta*) from two regions of Thailand. Int J Food Microbiol 128: 197–202. 18819720 10.1016/j.ijfoodmicro.2008.08.005

[38] Sørensen LM, Lametsch R, Andersen MR, Nielsen PV, Frisvad JC (2009) Proteome analysis of *Aspergillus niger*: Lactate added in starch-containing medium can increase production of the mycotoxin fumonisin B_2_ by modifying acetyl-CoA metabolism. BMC Microbiol 9: 255. 20003296 10.1186/1471-2180-9-255 PMC2807875

[39] Moore GG, Elliott JL, Singh R, Horn BW, Dorner JW, et al (2013) Sexuality generates diversity in the aflatoxin gene cluster: evidence based on a global scale. PLOS Path 9: e1003574. 10.1371/journal.ppat.1003574 PMC3757046 24009506

[40] Mogensen JM, Varga J, Thrane U, Frisvad JC (2009) *Aspergillus acidus* from Puerh tea and black tea does not produce ochratoxin A and fumonisin B_2_ . Int J Food Microbiol 132: 141–144. 19439385 10.1016/j.ijfoodmicro.2009.04.011

[41] Noonim P, Mahakarnchanakul W, Nielsen KF, Frisvad JC, Samson RA (2009) Fumonisin B_2_ production by *Aspergillus niger* from Thai coffee beans. Food Addit Contam 26: 94–100. 10.1080/02652030802366090 19680876

[42] Frisvad JC, Smedsgaard J, Samson RA, Larsen TO, Thrane U (2007) Fumonisin B_2_ production by *Aspergillus niger* . J Agric Food Chem 55: 9727–9732. 17929891 10.1021/jf0718906

[43] Jørgensen TR, Nielsen KF, Arentshorst M, Park J, van den Hondel C, et al (2011) Submerged conidiation and product formation by *Aspergillus niger* at low specific growth rates are affected in aerial development mutants. Appl Environ Microbiol 77: 5270–5277. 21652743 10.1128/AEM.00118-11 PMC3147447

[44] Frisvad JC, Larsen TO, de Vries R, Meijer M, Houbraken J, et al (2007) Secondary metabolite profiling, growth profiles and other tools for species recognition and important *Aspergillus* mycotoxins. Stud Mycol 59: 31–37. 18490955 10.3114/sim.2007.59.04 PMC2275202

[45] Ferracin LM, Frisvad JC, Taniwaki MH, Iamanaka BT, Sartori D, Schaoavaloff ME, Fungaro MHP (2009) Genetic relationships among strains of the *Aspergillus niger* aggregate. Braz Arch Biol Technol 52: 241–248.

[46] Hocking AD, Pitt JI (1980) Dichloran-glycerol medium for enumeration of xerophilic fungi from low-moisture foods. Appl Environ Microbiol 39: 488–492. 7387151 10.1128/aem.39.3.488-492.1980 PMC291365

[47] Frisvad JC, Samson RA (2004) Polyphasic taxonomy of *Penicillium* subgenus *Penicillium*. A guide to identification of food and air-borne terverticillate Penicillia and their mycotoxins. Stud Mycol 49: 1–173.

[48] Nielsen ML, Nielsen JB, Rank C, Klejnstrup ML, Holm DMK, et al (2011) A genome-wide polyketide synthase deletion library uncovers novel genetic links to polyketides and meroterpenoids in *Aspergillus nidulans* . FEMS Microbiol Lett 321: 157–166. 21658102 10.1111/j.1574-6968.2011.02327.x

[49] Boehm J, Hoff B, O'Gorman CM, Wolfers S, Klix V, et al (2013) Sexual reproduction and mating-type mediated strain development in the penicillin-producing fungus *Penicillium chrysogenum* . Proc Nat Acad Sci USA 110: 1476–1481. 23307807 10.1073/pnas.1217943110 PMC3557024

[50] Houbraken J, Frisvad JC, Samson RA (2011) Fleming's penicillin producing strain is not *Penicillium chrysogenum* but *P. rubens* . IMA Fungus 2: 87–95. 22679592 10.5598/imafungus.2011.02.01.12 PMC3317369

[51] Smedsgaard J (1997) Micro-scale extraction procedure for standardized screening of fungal metabolite production in cultures. J Chromatogr A 760: 264–270. 9062989 10.1016/s0021-9673(96)00803-5

[52] Frisvad JC, Thrane U (1987) Standardized High-Performance Liquid Chromatography of 182 mycotoxins and other fungal metabolites based on alkylphenone indices and UV-VIS spectra (diode-array detection). J Chromatogr 404: 195–214. 3680432 10.1016/s0021-9673(01)86850-3

[53] Houbraken J, Spierenburg H, Frisvad JC (2012) *Rasamsonia*, a new genus for thermotolerant and thermophilic *Talaromyces* and *Geosmithia* species. Antonie van Leeuwenhoek 101: 403–421. 21965082 10.1007/s10482-011-9647-1 PMC3261388

[54] Hong S-B, Lee M, Kim D-H, Varga J, Frisvad JC, et al (2013) *Aspergillus luchuensis*, an industrially important black *Aspergillus* in East Asia. PLOS ONE 8: e63769. 23723998 10.1371/journal.pone.0063769 PMC3665839

[55] Perrone G, Stea G, Epifani F, Varga J, Frisvad JC, et al (2011) *Aspergillus niger* contains the cryptic phylogenetic species *A. awamori* . Fungal Biol 115: 1138–1150. 22036292 10.1016/j.funbio.2011.07.008

[56] Bradshaw B, Etxebarria-Jardi G, Bonjoch J (2008) Polycyclic framework synthesis of anominine and tubingensin A indole diterpenes. Org Biomol Chem 6: 772–778. 18264578 10.1039/b718280e

[57] Staub GM, Gloer JB, Wicklow DT, Down PF (1992) Aspernomine: a cytotoxic antiinsectan metabolite with a novel ring system from the sclerotia of *Aspergillus nomius* . J Amer Chem Soc 114: 1015–1017.

[58] Sørensen A, Lübeck PS, Lübeck M, Nielsen KF, Ahring BK, et al (2011) *Aspergillus saccharolyticus* sp. nov., a new black *Aspergillus* species isolated in Denmark. Int J Syst Evol Microbiol 61: 3077–3083. 21335500 10.1099/ijs.0.029884-0

[59] Fennell DI, Warcup JH (1959) The ascocarps of *Aspergillus alliaceus* . Mycologia 51: 409–415.

[60] Gloer JB (1995) Antiinsectan natural products from fungal sclerotia. Acc Chem Res 28: 343–350.

[61] Agnihotri VP (1968) Effects of nitrogenous compounds on sclerotium formation in *Aspergillus niger* . Can J Microbiol 14: 1253–1258. 5724894 10.1139/m68-208

[62] Agnihotri VP (1969) Some nutritional and environmental factors affecting growth and production of sclerotia by a strain of *Aspergillus niger* . Can J Microbiol 15: 835–840. 5344736 10.1139/m69-149

[63] Kjer J, Debbab A, Aly AH, Proksch P (2010) Methods for isolation of marine-derived endophytic fungi and their bioactive secondary products. Nat Protocols 5: 479–490. 20203665 10.1038/nprot.2009.233

[64] Frisvad JC (2012) Media and growth conditions for induction of secondary metabolite production. Meth Mol Biol 944: 47–58. 10.1007/978-1-62703-122-6_3 23065607

[65] Mendes JAS, Prozil SO, Evtuguin DV, Lopes LPC (2013) Towards comprehensive utilization of winemaking residues: Characterization of grape skins from red grape pomaces of variety Touriga Nacional. Ind Crop Prod 43: 25–32.

[66] Myung K, Parobek AP, Godbey JA, Bowling AJ, Pence HE (2013) Interaction of organic solvents with the epicuticular wax layer of wheat leaves. J Agric Food Chem 61: 8737–8742. 23964787 10.1021/jf402846k

[67] Rank C, Klejnstrup ML, Petersen LM, Kildegaard S, Frisvad JC, Gotfredsen CH, Larsen TO (2012) Comparative chemistry of *Aspergillus oryzae* (RIB40) and *A. flavus* (NRRL 3357). Metabolites 2: 39–56. 24957367 10.3390/metabo2010039 PMC3901201

