# Supplementary material for: Formation of Sclerotia and Production of Indoloterpenes by Aspergillus niger and Other Species in Section Nigri
Source: PLoS One. 2014 Apr 15;9(4):e94857. doi: 10.1371/journal.pone.0094857 (PMC3988082; doi:10.1371/journal.pone.0094857)
Supplement: File S1 — Table S1. Table S1 is a list of fruits or seeds used to induce sclerotium production in Aspergillus niger strains. Table S2. Table S2 is a list of isolates in Aspergillus brasiliensis, A. niger and A. welwitchiae that do not produce sclerotia, even when stimulated by whole raisins in CYA agar in addition to a pre-freezing step. (DOCX) [file pone.0094857.s001.docx]

Supplementary material

Formation of sclerotia and production of indoloterpenes by *Aspergillus niger* and other species in section *Nigri.*

Jens C. Frisvad, Lene M. Petersen, E. Kirstine Lyhne, and Thomas O. Larsen

**Table S1**. List of fruits or seeds used to induce sclerotium production in *Aspergillus niger* strains.

| Plant part* | Type | Brand | Country of origin | Imported to |
| --- | --- | --- | --- | --- |
| Raisins | Dark | Sun-Maid | California, USA | Denmark |
| Sultanas raisins | Dark, organic | Unknown | Turkey | Urtekram, Denmark |
| Raisins | Dark | Unknown | Turkey | Urtekram, Denmark |
| Raisins | Dark, organic | Unknown | Argentina | Biogan A/S, Denmark |
| Raisins | Dark, organic | Unknown | California, USA | Biogan A/S, Denmark |
| Raisins | Dark, organic | Naturata | Slovenia | Bought in Slovenia |
| Raisins | Dark, organic | Unknown | California, USA | Helios A/S, Denmark |
| Raisins | Dark, organic | Sun-Maid | California, USA | Denmark |
| Raisins | Dark | Frutexa | Chile | Denmark |
| Sultana raisins | Dark | Unknown | Unknown | X-tra, COOP, Denmark |
| Jumbo raisins | Dark | Delicata | Unknown | System Frugt A/S, Tilst, Denmark |
| Raisins (two batches) | Dark | Unknown | Turkey | Irma, COOP, Denmark |
| Raisins | Dark, organic | Thompson | California, USA | Irma, COOP, Denmark |
| Raisins | Green | Thompson | California, USA | Denmark |
| Raisins | Green | Unknown | Unknown | System Frugt A/S, Tilst, Denmark |
| Small dry apricots | Stoneless, organic | Unknown | Unknown | Biogan A/S, Denmark |
| Small dry prunes | Stoneless, organic | Unknown | California, USA | Helios, Denmark |
| Dry goji berries |  |  | China | Superfruit, Sweden then to Denmark |
| Dry craneberries | 60%, apple juice, sunflower oil | Unknown | Unknown | System Frugt, Denmark |
| Dry red kidney beans | organic | Unknown | Unknown | Urtekram, Denmark |
| Dry white mulberries | Wild-crafted and sundried | Unknown | Asia | Superfruit / Rich Nature, Denmark, batch S090225-0410-160 |
| Dry blueberries | 55%, with apple juice and sunflower oil | Unknown | USA | Superfruit, Sweden then to Denmark |
| Dry green coffee | Arabica | Unknown | Brazil | Denmark |
| Black pepper |  | Unknown | Indonesia | Denmark |
| Rice | white | Uncle Ben | USA | Denmark |
| Rice | Brown, long grain | Organic Cuisine | Unknown | Nordic Food Partners A/S, Denmark |
| Corn | Canned | Unknown | USA | Denmark |
| Mango | Fresh | Unknown | Brazil | Denmark |
| Papaya | Fresh | Unknown | Brazil | Denmark |
| Sunflower oil | Organic, cold-pressed | Unknown | Unknown | Produced in the Netherlands, then imported to Denmark |

*All raisins were added up to 0.5% sunflower oil according to the producer. All fruits were placed as whole fruits / seeds in the same pattern as a three point inoculation of fungal colonies. The fungi were inoculated just beside the fruit parts (approximately 2 mm from the fruits). The rice was suspended in water and autoclaved. An approximately 10^7^ conidia / ml suspension was used for inoculation of the rice. Pieces of mango and papaya peel (approximately 1 x 2 cm were also placed in the CYA plates in three points before inoculation. For the 4% and 40% macerated mango and papaya in agar, both fruit flesh and peel was used, but the kernel / seeds were discarded.

**Table S2**. *Aspergillus* section *Nigri* List of isolates in *Aspergillus* *brasiliensis*, *A. niger* and *A. welwitchiae* that do not produce sclerotia even when stimulated by whole raisins in CYA agar in addition to a pre-freezing step.

| Species | Isolates with no sclerotia |
| --- | --- |
| *A. brasiliensis* | IBT 28083 = CBS 246.65  IBT 21946 = CBS 101740 |
| *A. niger* | IBT 27878 = NRRL 328, IBT 24637, IBT 29899 = NRRL 363, IBT 29884 = NRRL 4757, IBT 29885 = NRRL 612, IBT 28099 = CBS 139.52, IBT 23540 = NRRL 3112, IBT 21853, IBT 23680 = IMI 041871, IBT 29887 = NRRL 611, IBT 24634 = CBS 133817, IBT 29709 = CBS 513.88, IBT 27876 = NRRL 326, IBT 24637, NRRL 2372 = IBT 29890, NRRL 328 = IBT 27878, CBS 119725 = IBT 28098, NRRL 593 = IBT 29891, IBT 29021, IBT 29023, IBT 29002, IBT 29004 |
| *A. welwitchiae* (“*A. awamori*”), phylospecies | IBT 26343 = CBS 102.12; IBT 26387 = NRRL 567; IBT 26392 = NRRL 2001; IBT 3277 = CBS 618.78; IBT 28086 = ITEM 7097; IBT 28861 = NRRL 320; IBT 29098; IBT 29882 = NRRL 362; IBT 29894 = NRRL 595; IBT 29881 = NRRL 4851; IBT 29879 = NRRL 340; IBT 29888 = NRRL 6408; IBT 29890 = NRRL 372; IBT 29895 = NRRL 604 |
